# Reduced olfactory acuity in recently flightless insects suggests rapid regressive evolution

**DOI:** 10.1186/s12862-022-02005-w

**Published:** 2022-04-16

**Authors:** Stefanie Neupert, Graham A. McCulloch, Brodie J. Foster, Jonathan M. Waters, Paul Szyszka

**Affiliations:** Department of Zoology, University of Otago, Dunedin, 9054 New Zealand

**Keywords:** Regressive evolution, ‘Use it or lose it’, Evolutionary lability, Energetic cost, Selective pressure, Flight, Temporal resolution, Olfactory acuity, Plecoptera, Electroantennogram

## Abstract

**Background:**

Insects have exceptionally fast smelling capabilities, and some can track the temporal structure of odour plumes at rates above 100 Hz. It has been hypothesized that this fast smelling capability is an adaptation for flying. We test this hypothesis by comparing the olfactory acuity of sympatric flighted versus flightless lineages within a wing-polymorphic stonefly species.

**Results:**

Our analyses of olfactory receptor neuron responses reveal that recently-evolved flightless lineages have reduced olfactory acuity. By comparing flighted versus flightless ecotypes with similar genetic backgrounds, we eliminate other confounding factors that might have affected the evolution of their olfactory reception mechanisms. Our detection of different patterns of reduced olfactory response strength and speed in independently wing-reduced lineages suggests parallel evolution of reduced olfactory acuity.

**Conclusions:**

These reductions in olfactory acuity echo the rapid reduction of wings themselves, and represent an olfactory parallel to the convergent phenotypic shifts seen under selective gradients in other sensory systems (e.g. parallel loss of vision in cave fauna). Our study provides evidence for the hypothesis that flight poses a selective pressure on the speed and strength of olfactory receptor neuron responses and emphasizes the energetic costs of rapid olfaction.

**Supplementary Information:**

The online version contains supplementary material available at 10.1186/s12862-022-02005-w.

## Background

The origin of flight posed novel challenges for animals’ sensory systems, including the need for rapid processing of environmental information, because flying animals move faster and therefore experience more rapid changes in sensory stimuli. Accordingly, insects that fly faster, have evolved faster responding photoreceptor cells [1, 2]. This need for rapid sensing seems to be particularly pronounced for olfaction [3, 4], because both the speed of wind-borne odour plumes and the rate of odour concentration fluctuations increase with increasing distance from the ground [5, 6]. Indeed, olfactory receptor neurons of flighted insects can respond to odorants rapidly (within 3 ms and with sub-millisecond precision) [7], they can resolve fast odorant fluctuations (above 100 Hz) [8–10], corollary discharge from flight motor circuits enhances the temporal resolution of the insect olfactory system [11], and flying fruit flies can identify and respond to odorants within just 85 ms [12]. While it seems obvious that flight must have generated selective pressure for rapid olfactory transduction (the transformation of olfactory stimuli into action potentials), there is still only little direct evidence with which to assess this hypothesis [4, 13–16]. This lack of research on the evolutionary lability of temporal acuity of olfaction stands in stark contrast to the many studies on the evolution of the specificity of olfaction, which demonstrate rapid evolutionary adaptation of the olfactory system to the animals’ chemical environments [13, 17–24].

While broad scale evolutionary trends in sensory systems are well documented, the pace at which such systems can evolve is poorly understood. Darwin [25] observed that loss of nonfunctional phenotypes (reductive evolution) is a repeated phenomenon in nature [26, 27]. Dramatic examples of such reductive evolutionary processes include rapid deterioration of inactivated genetic material (e.g. [28]), and parallel losses of pigmentation and eyes in diverse cave fauna (e.g. [29, 30]). If maintenance of rapid olfactory transduction is key to the success of flighted insects, this begs the question of what happens to this sensory capacity when such lineages become secondarily wing-reduced. Indeed, flight loss has evolved independently in nearly every order of winged insects, and in some clades it has occurred repeatedly [31–33].

**Fig. 1:**
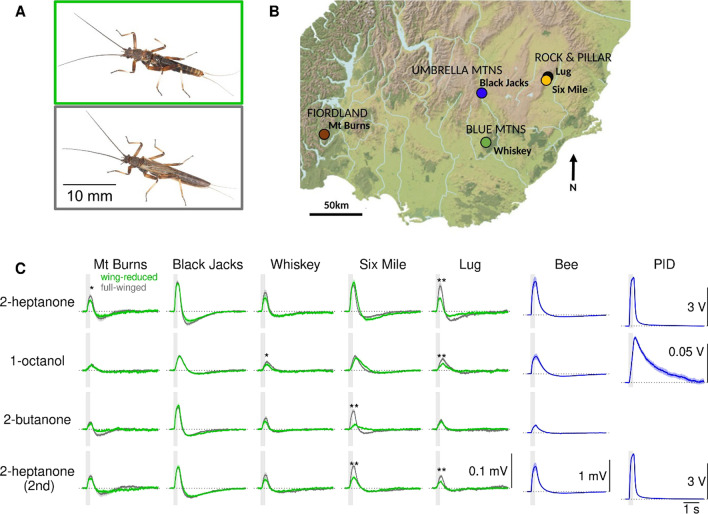
Odorant-evoked antennal responses are weaker in wing-reduced than in full-winged stoneflies. **A** Wing-reduced (green box) and full-winged (grey box) *Zelandoperla fenestrata* ecotypes. **B** Topographic map of the five sampling sites in New Zealand. **C** Antennal signal traces (mean ± SEM) for the five stonefly populations (grey: full-winged; green: wing-reduced). Number of antennae for wing-reduced/full winged stoneflies of the five populations (from left to right): 10/10, 24/23, 11/13, 11/14, 6/8. Signal traces of honey bee antennae 12 antennae) for scale and a photoionisation detector (PID) for visualizing the stimulus dynamics (10 recordings; PID signals for 2-butanone saturated and are not shown). Grey vertical bars indicate odorant valve opening time (300 ms). Horizontal dotted lines show 0 V

Under the ‘use it or lose it’ hypothesis, we propose that olfactory acuity becomes rapidly reduced in insect lineages that no longer use flight, in the same way that wings themselves become reduced. Here, we use a wing-dimorphic member of the early-diverging winged insect order Plecoptera as a model to test the hypothesis that rapid olfaction is a requirement specifically for flighted insects, but not for flightless lineages. The New Zealand *Zelandoperla fenestrata* Tillyard stonefly complex comprises both full-winged (flighted) and wing-reduced (non-flying) lineages that co-occur widely [34, 35] (Fig. 1A, B). Flight loss in *Z. fenestrata* is believed to be an evolutionary adaptation to high winds [36], with wing reduced populations typically found above the alpine treeline, or in lowland deforested regions [37–39]. Recent genomic analyses of this species complex indicate that wing reduction has evolved recently (likely within the last 15,000 years) and independently in different regions [37], and revealed differential expression of olfaction-related genes between full-winged versus wing-reduced stonefly ecotypes [39, 40]. Together, this makes *Z. fenestrata* a replicated model system for testing the hypothesis that flightless lineages exhibit secondarily reduced temporal olfactory acuity relative to that of flighted lineages.

## Results

To test the hypothesis that flight loss leads to a reduction of temporal olfactory acuity we compared odorant-evoked antennal responses between co-occurring full-winged and wing-reduced stonefly lineages (measured with electroantennogram recordings, EAG) (Fig. 1A). We sampled stoneflies from five genetically distinct stream populations [39] (Fig. 1B).

Antennal recordings in full-winged versus wing-reduced stonefly ecotypes revealed differences in the strength of antennal responses to odorants in four out of five stonefly populations (there was no difference in the Black Jacks population, Fig. 1C, Additional file 1: Fig. S1A). When differences in response strength were detected, wing-reduced stoneflies always showed weaker responses than full-winged stoneflies. Those weaker responses occurred for some but not all odorant stimuli, and odorants that evoked weaker responses in wing-reduced stoneflies, differed across stonefly populations. In the Six Mile population, wing-reduced stoneflies showed weaker responses to the second but not to the first set of 2-heptanone stimuli. This could indicate a faster deterioration or adaptation in antennae of wing-reduced stoneflies, however, we did not investigate the cause of this effect.

**Fig. 2:**
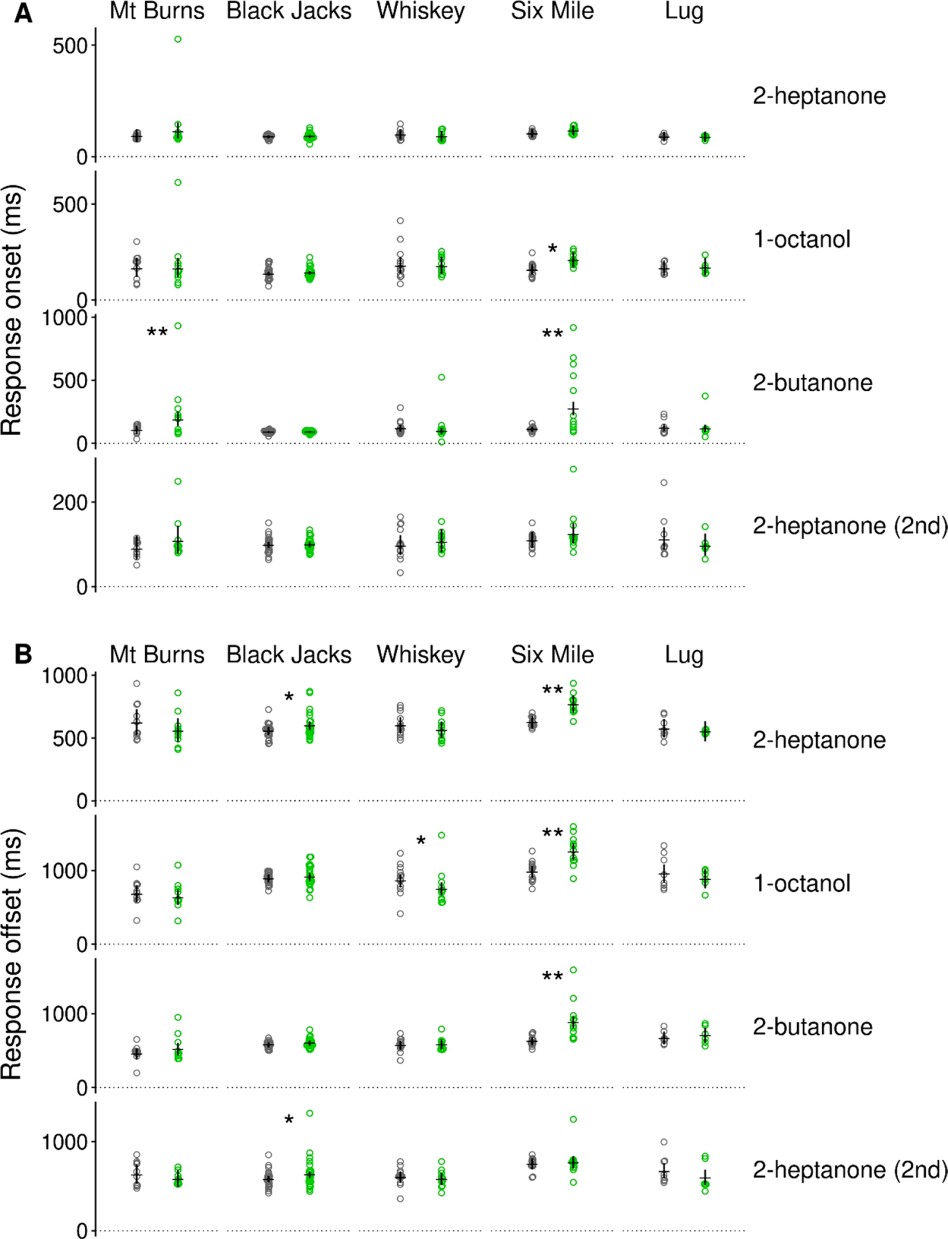
Odorant-evoked antennal responses are slower in wing-reduced than in full-winged stoneflies. **A** Antennal response onset time (time to 10% of signal maximum after valve opening, includes the time odorant need to reach the antenna), and **B** response offset time (time to 10% of signal maximum after the maximum). Grey: full-winged; green: wing-reduced. Circles show individual antennae. Horizontal black lines show means and vertical black lines show 95% credible intervals. * or ** greater than 95% or 99% certainty for differences between antennae of full-winged and wing-reduced stoneflies

In addition to weaker antennal responses, wing-reduced stoneflies showed slower response onset and offset times relative to full-winged stoneflies (Fig. 2A, B). Slower responses occurred in wing-reduced stoneflies from three out of five populations (Mt Burns, Black Jacks, Six Mile), they occurred for some but not all odorant stimuli, and odorants that evoked slower responses, differed across the different stonefly populations. In the Lug population, there were no differences in the response onset or offset times, and in the Whiskey population one odorant evoked a faster response offset time in wing-reduced stoneflies.

We probed the capability of stonefly antennae to resolve rapidly fluctuating odorant stimuli (Additional file 1: Fig. S1B) and recorded responses to 3-s long 10-Hz pulse series. However, antennae from neither full-winged nor wing-reduced stoneflies could track 10-Hz odorant pulses, indicating that antennal responses in stoneflies have a lower temporal resolution than antennal responses in more derived insect groups [9, 41, 42], including honey bees [8] (Additional file 1: Fig. S1B).

## Discussion

### Weaker and slower antennal responses in wing-reduced stoneflies

Wing-reduced stonefly ecotypes consistently displayed weaker antennal (EAG) responses and slower response onset and offset times relative to their full-winged counterparts. A weaker antennal response is associated with a lower action potential rate (within individual olfactory receptor neurons [43] or across a population of olfactory receptor neurons [44]), and a lower action potential rate reduces the temporal acuity of encoding the onset of an odorant stimulus [7, 45]. Therefore, the weaker antennal responses in wing-reduced individuals suggest these lineages have reduced temporal olfactory acuity.

The response dynamics of olfactory receptor neurons are shaped by multiple transduction (odorant-receptor (un)binding, receptor (de)activation), and adaptation processes, and the degree to which those processes contribute to a receptor neuron’s response dynamic depends on receptor type, odorant, and odorant dynamic [46–48]. Therefore, the finding that wing-reduced stoneflies from different streams show distinct, odorant-specific patterns of reduced response speed (Figs. 1C, 2) may indicate that those different transduction and adaptation processes were affected differently in the different lineages and suggests that these shifts evolved independently in each population. This inference is reinforced by recent genomic analyses, which indicate flight loss has independently evolved in each population [37, 39]. The current study thus provides additional evidence for the repeated evolution of flight loss, with different forms of reduced olfactory acuity detected in these independently flightless lineages.

Flightless *Z. fenestrata* stonefly ecotypes have evolved within the last 15,000 years [39], indicating the reduction of olfactory acuity has likewise occurred rapidly. Recent genomic studies indicate that clusters of the adaptive loci often underpin rapid adaptation [49–51]. If olfactory genes are closely genetically linked to the gene underpinning wing loss in *Z. fenestrata* this may facilitate parallel shifts in flight ability and olfaction. The exact genomic mechanism of wing loss in *Z. fenestrata* is not known, and likely varies across populations [39, 52]. However, a recent study suggests that the developmental supergene *doublesex* may play an important role, and this gene is closely physically linked to at least one odorant binding protein [39]. Future genomic studies promise to further unravel the potential role that genetic linkage may play in the parallel reductions of flight and olfactory ability in this species.

### Selective pressure for rapid olfactory transduction in flying insects

The faster onsets and offsets of odour-evoked antennal responses detected in full-winged stoneflies are likely to facilitate long-range search behaviour. When searching for an odorous target (e.g. a mating partner), flying insects encounter rapidly fluctuating odour plumes [53–55]. Because the randomness of air flow destroys most of the directional information present in an odour plume (e.g. concentration gradients), flying animals need to use reactive or infotactic strategies for odour plume tracking, both of which require the animal to rapidly detect and react to the target odour [56–60]. A fast onset of the antennal response would shorten the time to detect and react to the target odour, while fast offset of the antennal response would facilitate the detection of subsequent odour stimuli. In addition to facilitating odour plume tracking, rapid olfaction enables the perceptual segregation of mixed odorants from different sources based on short differences in the arrival of odorants [10, 61–66].

In line with the ecological requirement for rapid olfaction, insect olfactory receptor neurons can respond to odorants rapidly (within 3 ms) [7, 8]. Olfactory receptor neurons can respond rapidly because olfactory receptors are ion channels which are composed of odorant-specific olfactory receptors (OR) and co-receptors (Orco) [67–69]. Missbach et al. [14] suggested that the OR/Orco system represents a specific adaptation associated with insect flight (but see [13] for an opposing view). While OR genes likely evolved in the common ancestor of modern insects, perhaps as an adaptation to terrestrial conditions [13, 24], the OR/Orco system may have arisen more recently and may have facilitated rapid olfaction in winged insects [15]. The finding that even full-winged stoneflies had a relatively slow odorant pulse tracking capability (below 10 Hz; Additional file 1: Fig. S1B) compared to that of strong-flying insects (above 100 Hz [8]) may reflect the generally weaker flying ability of this early-diverging insect clade [70].

### Energetic costs of rapid olfactory transduction and mechanisms of its slackening

The reduced strength and speed of antennal responses in wing-reduced stoneflies suggests that olfactory transduction is energetically costly, which in turn generates a selective pressure for slackening olfactory transduction in flightless insects. The strength of an antennal (EAG) response correlates with the receptor current amplitude and action potential number [43, 44]. Because both ion currents and action potentials are energetically costly [71], the energetic cost of antennal responses increases with response strength. Likewise, a rapidly responding receptor neuron is likely to be energetically costly, because it requires a low membrane time constant which in turn, requires an energetically costly high membrane conductance [72].

Besides fast activation, high temporal resolution requires fast deactivation of olfactory receptor neurons, so that they can respond to following odorant pulses. Deactivation is thought to be mediated by removal of odorants from the OR/Orco complex through odorant-binding proteins and odorant-removing proteins [73]. A putative odorant-removing protein, *Pinocchio* [74], is significantly more expressed in the notum of wing-reduced than in full-winged stoneflies from Lug Creek [40]. If *Pinocchio* is similarly overexpressed in the antennae of wing-reduced stoneflies, this may explain the weaker odour-evoked antennal responses detected in wing-reduced stoneflies. Future comparisons of antenna morphologies, antennal expression of olfaction-related genes [75], and the number, membrane conductance, and action potential threshold of olfactory receptor neurons will further elucidate the mechanistic basis of this reduction in olfactory acuity.

## Conclusions

The findings of the current study highlight not only the need for rapid olfactory processing in flying insects [3], but also that olfactiory acuity can be rapidly reduced when no longer required (when flight ability is lost). The locally and independently wing-reduced lineages analysed here have diverged from their winged counterparts only very recently in evolutionary terms (during the current interglacial, less than 15,000 years ago) [37, 39]. This rapid reductive evolution of sensory ability echoes the rapid reduction of wings themselves, and also represents a neurobiological parallel to the rapid phenotypic shifts seen under sharp selective gradients in other systems (e.g. loss of vision in cave fauna) [29, 30]. Broadly, these findings emphasize the energetic costs of sensory acuity, and the key role of natural selection in shaping neurobiological shifts. Additionally, this multidisciplinary analysis highlights the potential for future studies to further elucidate evolutionary changes in sensory systems.

## Materials and methods

### Stonefly sampling

We sampled stoneflies from zones of ecotypic overlap from five genetically independent stream populations in New Zealand [37, 39]: Mount Burns, Black Jacks Creek, Whiskey Creek, Six Mile Creek and Lug Creek (Fig. 1B). Final instar nymphs were collected by hand from under stones or wood in stream cascades and rapids. Nymphs were subsequently reared in the laboratory in Styrofoam cups at 11 °C under a natural day:night cycle, in water from their natal stream. We collected nymphs of full-winged and wing-reduced ecotypes from the same locations, and reared them under the same conditions, so that any differences between them are unlikely to reflect environmental variation. After emerging as adults, stoneflies were sexed based on genitalia, and morphologically characterized as either full-winged or wing-reduced.

### Antennal responses to olfactory stimuli

We used electroantennograms (EAGs) of detached antennae as a proxy for the amplitude and dynamics of olfactory receptor neuron responses. The amplitude of EAG signals increases with increasing receptor current amplitudes and with increasing number of activated receptor neurons [43, 44, 76]. Note that EAG signals do not accurately reflect spike rates, but there is a positive correlation between receptor current amplitude and spike rate [43, 46, 77]. We chose EAG recordings over single neuron recordings, because we could not collect the high number of stoneflies that would have been needed for single neuron recordings. For the experiments shown in Figs. 1, 2 and Additional file 1: S1, we used 1- to 5-day old adult male stoneflies and female honey bees. For the experiments shown in Additional file 1: Fig. S2 we used male and female stoneflies. We used a razor blade to cut off a 5 mm long section of the distal antennae. Antennae were mounted with conductive gel (GEL+, Ritex, Germany) on a four-channel silver electrode [66]. Five minutes later, the antennae were placed at a distance of 2 mm in front of the outlet of the olfactory stimulator. To eliminate between-session variability (e.g., due to humidity or circadian rhythms in antennal responsiveness [78]), the left and right antennae of one full-winged and one wing-reduced stonefly were recorded simultaneously. EAG signals were differentially amplified against the reference electrode using 1000× gain, recorded in AC-coupled mode and low-pass-filtered at 1 kHz (MA 103 preamplifier and MA 102 four-channel amplifier, Universität zu Köln). The distal tips of the four antennae were mounted on a common central electrode that was connected to the inverted inputs of the preamplifiers. Under this approach, positive EAG signals reflect excitatory responses (activation of olfactory receptor neurons) and negative EAG signals reflect inhibitory responses.

### Olfactory stimulation

We used a custom-made 6-channel olfactory stimulator (same approach as in [79] but built with materials as described in [80]). This stimulator provided a constant airflow (volume flow rate = 4.8 L/min, flow speed = 100 cm/s). The antennae were exposed to this constant airflow throughout the whole duration of the recordings before and between the odorant stimuli. To apply odorants, odorant-laden air (300 mL/min) was injected into the constant carrier air stream and, simultaneously, the same amount of clean air was withdrawn to keep the total airflow constant. The odorant air stream was produced by bottled compressed air and the carrier air stream was produced by an aquarium pump. Both air streams were filtered with active carbon filters (HN4S-AUN, Parker). The stimulation was controlled with the data acquisition system Micro 3 1401 and Spike2 software version 8.03 (CED).

All odorants were kept in glass vials with PTFE septum screw caps (20 mL EPA vial, JG Finneran). We presented the following olfactory stimuli in the following sequence: odourless blank (empty vial), 2-heptanone, 1-octanol, and 2-butanone (these three odorants purchased from Sigma-Aldrich). We chose the odorants for the following reasons: 2-heptanone because it induces robust antennal responses in different insect species [8]; 1-octanol because it was used in a previous EAG study of stoneflies [81], 2-butanone because of its fast stimulus dynamics [61]. We also trialed propionic acid (Sigma-Aldrich), which had evoked particularly strong antennal responses in a previous study [81], and we tested water, but we ultimately excluded these two odorants following the detection of non-biological artifacts (Additional file 1: Fig. S2).

We used a photoionisation detector (miniPID B, Aurora) to measure the dynamics of odorant concentration change (Fig. 1C, Additional file 1: Fig. S1B). To vary the strength of olfactory stimuli we presented each odorant at up to four different pulse durations (15, 30, 150 and 300 ms valve open time, Additional file 1: Fig. S1A). Because the rise time of the odorant concentration was larger than 150 ms, these different stimulus durations resulted in different maximum concentrations. Note that odorant-specific differences in stimulus dynamics are a consequence of interactions between odorants and the surfaces of both the olfactory stimulator and the photoionisation detector [47, 80]. We also recorded antennal responses in honey bees (Fig. 1C, Additional file 1: Fig. S1) as a scale for comparing stoneflies’ antennal responses to other insects (see temporal resolution of antennal responses of honey bees, locusts, moths, and cockroaches in a previous study [8]).

Each olfactory stimulus (odorant/pulse duration combination) was presented ten times at an inter-stimulus interval of 5 s. Ten seconds after the last 300 ms long stimulus we presented fluctuating 10-Hz stimuli by repetitively opening the valve for 50 ms at a frequency of 10 Hz over a period of 3 s (Additional file 1: Fig. S1B). This 10-Hz stimulus served to quantify the temporal resolution. Five seconds after the 10-Hz stimulus the next odorant started. At the end of each experiment we presented another 10 stimuli of 2-heptanone (300 ms) to test whether the antennae were still responding. Stimulus protocols varied between stream populations in the number of stimuli, and these differences could explain differences in antennal responses between stream populations. We excluded 22 antennae that showed sudden baseline shifts.

### Data analysis

EAG signal data were exported from Spike2 and then further analyzed using R (version 3.6.3) [82]. Each recording was cut into 5 s traces, starting 0.2 s before and ending 4.8 s after each stimulus onset. Each trace was baseline-corrected by subtracting the median voltage during the 0.2 s time window before valve opening from the entire trace. To increase the signal-to-noise ratio, we calculated the median trace over each set of 10 stimulations with the same odorant/pulse duration combination. To reduce the noise, we applied a running median filter with a window size of 11 ms on the traces.

To assess whether an antenna shows stimulus-induced responses to the last set of 2-heptanone stimulations, we defined a response threshold as two times the standard deviation of the last 3 s of the trace. If the stimulus induced response of an antenna to these 2-heptanone stimulations did not exceed the response threshold for at least 50 consecutive ms between 50 and 1000 ms after valve opening, we excluded all recordings from this antenna from further analysis (68 of 76 full-winged antennae and 62 of 77 wing-reduced antennae were analyzed).

We quantified different response parameters evoked by 2-heptanone, 1-octanol, and 2-butanone between stonefly ecotypes for a given odorant and collection site: (1) response strength as the mean response in the time window of 25 ms before and 25 ms after the maximum signal between 50 ms and 1 s after valve opening; (2) response onset: time between valve opening and 10% of maximum signal (before signal maximum); (3) response offset time: time between valve opening and 10% of maximum signal (after signal maximum); and (4) temporal resolution for 10-Hz stimulations: power spectral densities on a 3 s time window starting 0.4 s after the first valve opening using the *multitaper* R package[83] with the *sine taper* method.

To quantify if there are differences in response strength between ecotypes, we ran linear mixed models with log2 transformed response strength as the response variable. To avoid negative values, we added an offset of 0.01 to each mean response prior to the log2 transformation. We included ecotype (full-winged or wing-reduced) and pulse duration as explanatory variables and added an interaction between both variables. To account for repeated measurements of the same antennae, we included antenna identity as a random factor.

To test if there are differences in response timing, i.e. in response onset time or offset time between stonefly ecotypes for a given odorant and collection site, we ran linear models. We included the log2 transformed response timing of interest (either response onset, or response offset) for 300 ms long pulses as the response variable and ecotype (full-winged or wing-reduced) as explanatory variable.

Inferences for all types of models were drawn using Bayesian statistics. We used an improper prior distribution (flat prior) and simulated 10,000 random draws from the posterior distribution using the function *sim* from the R package *arm* [84].

We used model estimates as the mean and the 2.5% and 97.5% quantiles as the lower and upper limit of the 95% credible interval. We calculated the proportion of simulated values from the posterior distribution that are bigger for one ecotype (e.g. A) over the other (e.g. B). A resulting proportion of 0.99 would mean that we are 99% certain that ecotype A has a larger parameter value than ecotype B. For plotting, we back-transformed the quantiles in the original scale. For the mean response strength, we subtracted the offset of 0.01 again. We marked differences between full-winged and wing-reduced ecotypes in a given odorant/pulse duration configuration of > 95% and > 99% certainty with one and two asterisks, respectively.

## Supplementary Information

**Additional file 1: Figure S1.** Antennal responses to different stimulus durations and pulse series. **Figure S2.** Biological and non-biological antennal signals are independent from each other.

## Data Availability

The datasets generated and analysed during the current study are available at 10.12751/g-node.yq9qml.

## Abbreviations

EAG: Electroantennogram
OR: Olfactory receptor
Orco: Olfactory receptor co-receptors
PID: Photoionisation detector
