# Correction to: Reduced olfactory acuity in recently flightless insects suggests rapid regressive evolution

**DOI:** 10.1186/s12862-022-02013-w

**Published:** 2022-05-02

**Authors:** Stefanie Neupert, Graham A. McCulloch, Brodie J. Foster, Jonathan M. Waters, Paul Szyszka

**Affiliations:** Department of Zoology, University of Otago, Dunedin, 9054 New Zealand

## Correction to: BMC Ecology and Evolution (2022) 22:50 10.1186/s12862-022-02005-w

Following the publication of the original article [1], we were notified that the incorrect additional file had been linked to the article.

The original article has been [1] corrected.
