# Supplementary material for: Reduced olfactory acuity in recently flightless insects suggests rapid regressive evolution
Source: BMC Ecol Evol. 2022 Apr 16;22:50. doi: 10.1186/s12862-022-02005-w (PMC9013461; doi:10.1186/s12862-022-02005-w)
Supplement: Supplementary file 1 — Additional file 1: Figure S1. Antennal responses to different stimulus durations and pulse series. Figure S2. Biological and non-biological antennal signals are independent from each other. [file 12862_2022_2005_MOESM1_ESM.pdf]

Additional Information

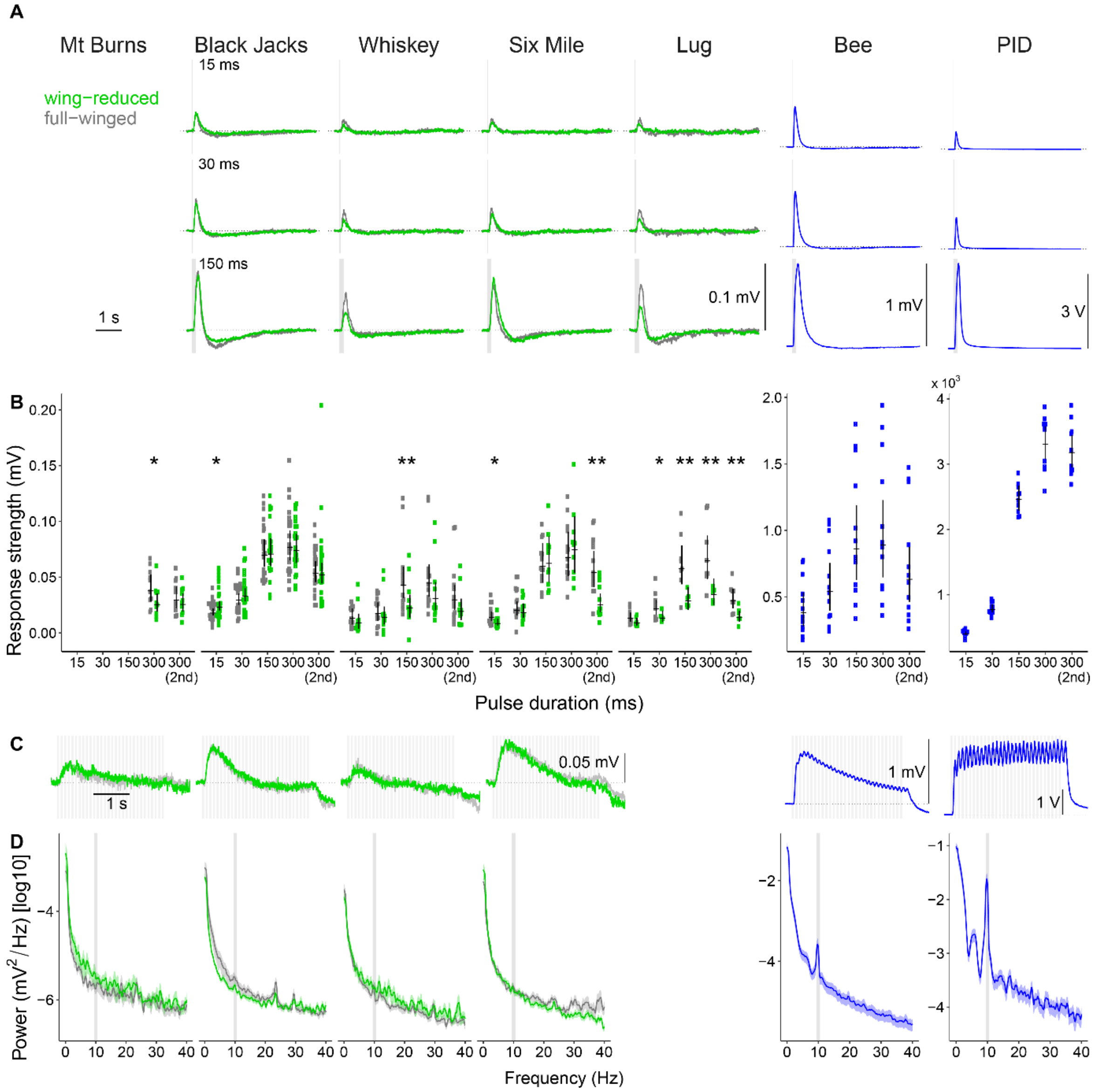

Fig. S1. Antennal responses to different stimulus durations and pulse series. (A) Antennal signal traces (mean ± SEM) of the same antennae as in Fig. 1C, but during stimulation with 15, 30, and 150 ms pulses of 2-heptanone (Mt Burns stonefly antennae were not stimulated with those pulse durations). Grey vertical bars indicate odorant valve opening times. Horizontal dotted lines show 0 V. **(B)** Response strength during stimulation with 2-heptanone at different pulse durations. Horizontal black lines show means and vertical black lines show 95% credible intervals. * or **: greater than 95% or 99% probability for differences between antennae of full-winged (grey) and wing-reduced (green) stoneflies. Circles show individual antennae. **(C)** Median signal traces for stonefly and honey bee antennae and photoionisation detector (PID) during 3 seconds-long 10-Hz pulse series (Lug stonefly antennae were not stimulated with 10-Hz pulse series). **(D)** Power spectral densities (mean ± SEM) for the same recordings as in (C). The lack of peaks at 10 Hz indicates that the antennal responses of stoneflies could not resolve 10-Hz odorant pulses. The peak at 10 Hz for antennal responses of honey bees show that they can resolve 10-Hz odorant pulses.

Additional Methods

Testing the effect of non-biological electrical antenna properties on antennal responses.

The strength of an antennal response can be affected by the physical properties of the antenna itself (1). Therefore, the weaker antennal responses in wing-reduced individuals could reflect a difference in the antennae's non-biological physical properties. To test whether weaker antennal responses in wing-reduced stoneflies could reflect non-biological differences between antennae, we recorded antennal responses in live versus dead antennae (Fig. S2A).

To identify any potential non-biological EAG signals, we recorded 9 additional stonefly antennae (from 5 male and 4 female full-winged individuals from Six Mile). After the recording, we killed the antennae by applying 90°C hot water vapor for 2 to 5 min prior to another set of EAG recordings with the same stimulus protocol.

To induce antennal responses in these live and dead antennae, we applied blank (empty vial), 2-heptanone, 1-octanol, propionic acid, 2-butanone and water (same procedure as in the recordings from live antennae before). Live antennae responded to all odorants. Dead antennae responded to propionic acid and water, but not to the other odorants (Fig. S2A).

Therefore, while EAG signals evoked by propionic acid and water may include non-biological components, the other odorants assessed (2-heptanone, 1-octanol, 2-butanone) have no such artifacts. Propionic acid-evoked responses in dead antennae could be explained by dissociation of propionic acid when it comes in contact with water vapor in the air. This creates H^+ and OH^- ions, and uneven accumulation of these ions at the recording and reference electrodes would induce electrical potentials.

To test whether the signal strength of live antennae is dependent on their physical properties, we utilized the negative signals in dead antennae induced by propionic acid (physical property) and compared them to the response strength to 2-heptanone (because this odorant induced the strongest responses) of the same antennae, but when still alive (Fig. S2B). A linear relationship would indicate that the difference in response strength between full-winged and wing-reduced stonefly antennae could result from differences between the physical properties of antennae themselves. To test for such differences, we performed linear regression analyses for each of the different pulse durations (15, 30, 150, and 300 ms). We used the response strength to 2-heptanone in nine live antennae as a dependent variable and corresponding response strength to propionic acid of the same nine antennae when dead as an independent variable. We assessed whether the slope in each of the four regression analyses were significantly different from zero. The finding that the strength of odorant-evoked signals in live antennae did not depend on the signal strength evoked in dead antennae (Fig. S2B) confirms that the differences in antennal responses between wing-reduced and full-winged stoneflies reflect true biological differentiation. Therefore, the weaker and slower antennal responses in wing-reduced individuals confirm that flightless lineages have reduced olfactory acuity.

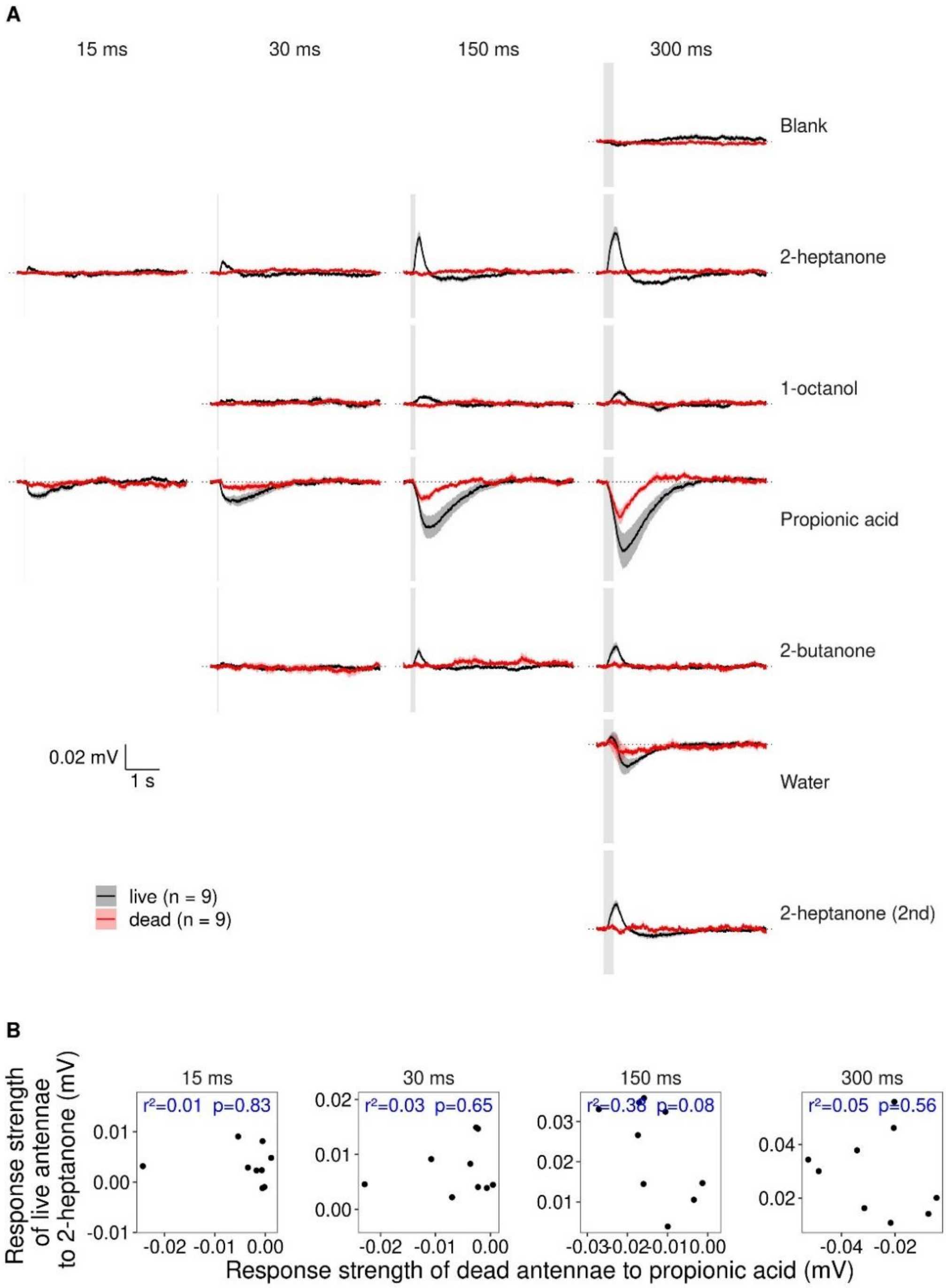

Fig. S2. Biological and non-biological antennal signals are independent from each other. (A) EAG signal traces (mean ± SEM, N = 9 antennae) of live (black) and dead (red) antennae (full-winged stoneflies from Six Mile Creek, dead and alive antennae were identical) during stimulation with different odorants (rows) and pulse durations (columns). Grey vertical bars indicate valve opening time. The sequence of panels (top left to bottom right) corresponds to the sequence of stimuli. **(B)** Scatter plot of signal strength in dead antennae to propionic acid in relation to their signal strength to 2-heptanone when these antennae were still alive. Each dot per panel shows an individual antenna. Blue values show the proportion of variance (r^2) explained and the p-value (p) for non-zero association between dependent and independent variable.

Reference

1. Kapitskii S V., Gribakin FG. Electroantennogram of the American cockroach: effect of oxygen and an electrical model. J Comp Physiol A. 1992 Jun;170(5):651–63.
